# Autopsy findings of fatal retroperitoneal hemorrhage after traumatic rupture of bilateral renal angiomyolipoma

**DOI:** 10.4322/acr.2024.482

**Published:** 2024-03-15

**Authors:** Enrico De Dominicis, Gian Luca Marella, Gabriele Giuga, Giulia Ceccobelli, Luca Savino, Alessandro Mauro Tavone

**Affiliations:** 1 ASL Frosinone, UOC Medicina Legale Territoriale, Frosinone, Lazio, Italy; 2 University of Rome Tor Vergata, Department of Surgical Sciences, Rome, Italy; 3 University of Rome Tor Vergata, Department of Biomedicine and Prevention, Rome, Italy

**Keywords:** Retroperitoneal Space, Hemorrhage, Angiomyolipoma, Fatal Outcome

## Abstract

The present work reports the autopsy findings of a unique case characterized by fatal retroperitoneal hemorrhage following the traumatic rupture of bilateral renal angiomyolipomas. Renal angiomyolipomas are generally benign tumors with an unpredictable clinical course, ranging from asymptomatic to sudden rupture and hemorrhagic shock. They may be associated with genetic disorders such as tuberous sclerosis complex. The case under investigation is unprecedented in the medical literature due to its bilateral nature and fatal outcome. Autopsy analysis revealed an extensive retroperitoneal hemorrhage originating from bilateral ruptured tumors. Microscopic examination found features consistent with bilateral renal angiomyolipoma. Circumstantial information identified a traffic accident before the death, considering it as the cause of the tumors’ traumatic rupture. In this case, due to the severity of the situation, immediate medical measures—such as fluid resuscitation, coagulopathy correction, and surgical treatment, which are usually lifesaving—could not be performed. This led to the patient being declared dead at the scene of the crash.

## INTRODUCTION

Renal angiomyolipoma is a tumor originating from perivascular epithelial cells in the PEComa family. It can be classified into two types based on its composition: (i) the classic renal angiomyolipoma, which consists of thick dysmorphic blood vessels, smooth muscle, and adipose tissue, which rarely infiltrates the perirenal tissue, and (ii) a more aggressive variant with a more prominent fourth component, known as perivascular epithelioid cells.^1^

Angiomyolipomas may manifest as sporadic occurrences or be associated with tuberous sclerosis complex. Individuals with tuberous sclerosis complex often exhibit the classic triad of seizures, adenoma sebaceum, and cognitive impairment, with renal angiomyolipomas developing in 80% of them. It is now understood; however, angiomyolipomas can also arise independently of tuberous sclerosis complex, occurring sporadically. Notably, a significant majority, around 80%, of those with angiomyolipomas do not have tuberous sclerosis complex.^2^

In 1993, Steiner et al.^2^ first showed with a long-term study that angiomyolipomas (AML) can grow and increase in dimension, and their size is the most reliable parameter for determining whether surgery is necessary or a conservative approach can be adopted.

Due to the significant presence of adipose tissue in most cases, diagnosis is typically made using CT or MRI by identifying fat tissue within the mass,^3^ making it possible to distinguish between AMLs and renal cell tumors. However, further investigations are necessary to distinguish the specific histotype.

Symptoms caused by angiomyolipoma depend on its size. When symptomatic, embolization should be considered as rupture is a dreadful complication, and interventional therapies are necessary to control bleeding.^4^

Renal angiomyolipoma is the second most common cause of spontaneous renal rupture and perirenal hemorrhage after renal adenocarcinoma.^5^ Typically, the majority (64-77%) of tumors measuring less than 40 mm do not cause symptoms, whereas almost 90% of those larger than 40 mm exhibit symptoms.^4^ Symptomatic patients often present with the classical Lenks triad,^6^ which includes flank pain, a palpable tender mass, and signs of internal bleeding such as hematuria, intracapsular, or retroperitoneal hemorrhage. Other symptoms may include nausea, vomiting, fever, anemia, renal failure, and hypotension.^6-8^

The management of angiomyolipoma (AML) depends on its size and symptoms. Various therapeutic options are available, depending on the size of the tumor and the presence of symptoms:^3,4^ (i) tumor of ≤4 cm and asymptomatic should be followed every 12 months with ultrasound (US); (ii) small tumors with symptoms should be observed; however, arterial embolization or partial nephrectomy can be considered; (iii) large tumors without symptoms should be observed as the first choice, with US and computed tomography (CT); (iv) the management of symptomatic and large AML may vary due to Its vascularity - renal arterial embolization and partial nephrectomy is the treatment of choice. Also, transarterial ethanol or percutaneous ablation with radiofrequency or cryoablation may be used with unestablished results.

Herein, we present a case of a man who tragically died of hemorrhagic shock following the rupture of a completely asymptomatic and bilateral angiomyolipoma with massive retroperitoneal bleeding due to a car accident.

## CASE REPORT

A 37-year-old Caucasian male was found dead by emergency responders inside his car after a traffic accident. He was driving alone, and after losing control, he hit another vehicle and a tree on the side of the roadway.

Eyewitnesses reported the man bleeding from the head profusely and losing consciousness within a few minutes. Upon the arrival of medical first aid, the man was found with the driver’s seat belt unfastened, a wide laceration of the scalp, and the absence of an arterial pulse. The man was pronounced dead, and an autopsy was ordered.

## AUTOPSY PRESENTATION

The autopsy examination started 30 hours after the death. External examination revealed the face covered with blood and a “flap” laceration of skin in the left parietal region, measuring 13.5 cm, and with hemorrhagic edges infiltrations. Other small ecchymoses coexisted on the lower limbs. Since renal angiomyolipomas are present in tuberous sclerosis patients, especially with the bilateral presentation, skin and mucosal lesions were considered and excluded. Further head dissection revealed the absence of fractures on the vault and the base of the skull. A small subdural hemorrhage on the right parietal side without brain compression and no brain wounds were observed. No other kind of internal organs' lesions - including brain, lungs, and heart - were present.

**Figure 1:**
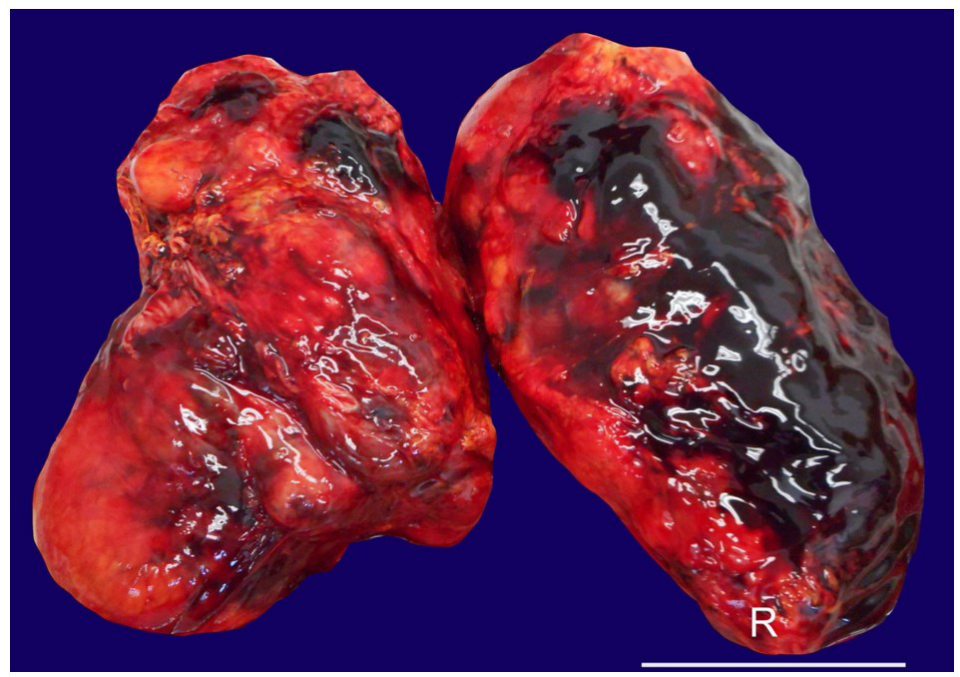
Gross view of both kidneys - A view of the kidneys following the removal of perirenal fat reveals the presence of extensive hemorrhagic infiltration on the surface of both kidneys, especially on the right one. Scale bar= 5 cm. (right kidney (R) exhibits the most extensive hemorrhagic component).

The initial dissection of the abdomen revealed a hemorrhagic infiltration of the mesentery radix without any traumatic wound of the abdominal organs. Further exploration of the retroperitoneal and renal spaces evidenced a bilateral perirenal blood collection (700 mL on the right and 250mL on the left) with hemorrhagic infiltration of the perirenal adipose tissue and the renal capsule, bilaterally (Figure 1).

**Figure 2:**
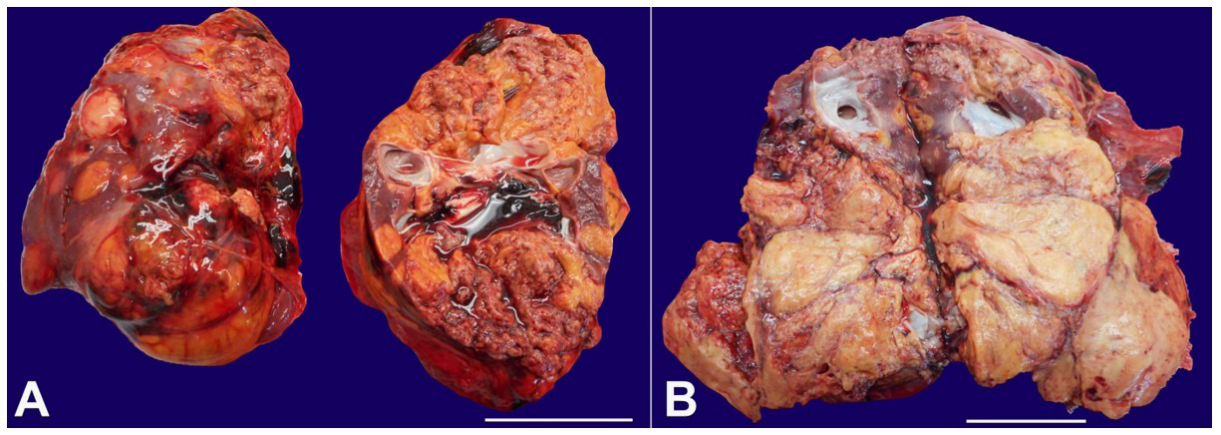
Macroscopic features of kidneys. **A –** The kidney surface is lobulated and enlarged with a brown-yellow color; **B –** The right kidney's cleavage surface shows that the parenchyma has been largely replaced by newly formed solid and yellowish tissue (scale bars= 5 cm).

After removing the renal capsules, both kidneys appeared increased, weighting 342 g the left and 389 g the right (men’s left kidney weight: average 160g, [range 50-410g]; men’s right kidney weight: average 162g, [range 53-320g],^9^ and solid, yellowish, multifocal, and bilateral nodules with a significant necrotic-hemorrhagic component infiltrating the renal calyxes substituted both renal parenchyma (Figures 2A and 2B).

**Figure 3:**
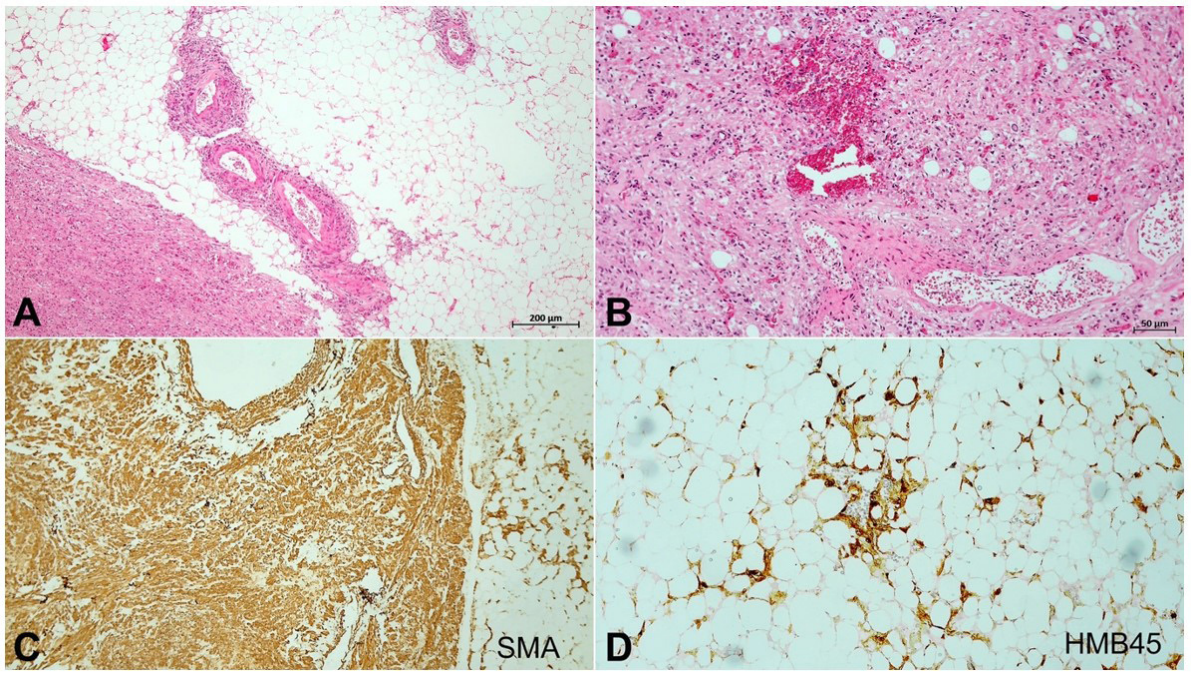
Photomicrographs of the kidney. **A** and **B –** Neoplastic tissue consisting of mature adipose tissue, with proliferation of smooth muscle tissue, newly formed vessels, and neoplastic cells with irregular nuclei increased of size (H&E original magnification 40x (A) and 100x (B)); **C –** Smooth muscle actin immunostaining confirmed the muscular component of the neoplasia (original magnification 40x); **D –** HMB45 immunohistochemistry staining was performed showing heterogeneous positivity in adipose cells, confirming the diagnosis of renal angiomyolipoma (original magnification 100x).

The microscopical examination of the renal samples rendered the diagnosis of bilateral angiomyolipoma, with blood congestion of the residual normal parenchyma (Figures 3A and 3B). Immunostaining confirmed the muscular and adipose components of the neoplasia (Figures 3C and 3D).

The remaining organs exhibited macroscopic and microscopic features associated with blood loss, such as marked pallor, poorly represented hypostasis, scant presence/absence of intravascular erythrocytes, and signs of terminal myocardial ischemia.

Further investigation with the man’s general practitioner confirmed that his medical history was silent and any kidney-related problem was unknown.

Finally, the medical examiner established that the man’s death was due to an acute hemorrhagic shock due to head trauma and traumatic rupture of the bilateral angiomyolipoma.

## DISCUSSION

According to the literature, there are three types of hemorrhagic etiologies concerning AML:^6-8,10^

Spontaneous retroperitoneal hemorrhage of non-traumatic origin called Wunderlich's Syndrome, which occurs in up to half of the patients with tumors larger than 40 mm;
AML rupture during pregnancy mimicking extra-uterine gravidity or other conditions;
Traumatic etiology: even after a minor traumatic event, when the renal AML can bleed or rupture.

In our case, there is no reason to suspect a different non-traumatic etiology related to the traffic accident.

Most angiomyolipomas contain substantial amounts of adipose tissue and are usually diagnosed using CT or MRI by identifying the imaging features of fat tissue within the tumor. Those that can be diagnosed using imaging are termed “Classic” angiomyolipomas.^11^ 80% of angiomyolipomas are sporadic and mostly inconsequential. Approximately 20% are associated with tuberous sclerosis complex.^12^ Angiomyolipomas may also be found associated with lymphangioleiomyomatosis (LAM).^13,14^

When an AML increases in size and becomes symptomatic, embolization should be considered. This is because rupture is a significant complication, and interventional therapies are required to halt bleeding.^1,15^

A retrospective study^16^ based on 23 patients with renal angiomyolipomas and tuberous sclerosis showed that the size of the angiomyolipomas correlates with the risk of bleeding. Renal angiomyolipomas larger than 3.5 cm in diameter carry a substantial risk for severe hemorrhage, necessitating follow-up every six months and a therapeutic approach.

Our case drew attention because of the bilaterally of the tumor. Bilateral renal angiomyolipoma is a rare entity most often associated with a genetic disorder, tuberous sclerosis complex, which is scarcely reported in the literature.^17,18^ It is probable that our patient did not know this eventual diagnosis. Renal AML traumatic rupture is rare but a documented finding in the literature.^19^

However, no deaths due to hemorrhage from traumatic rupture of a bilateral renal angiomyolipoma have been previously reported.

The present case shows 3 features that make it unique: (i) death due to hemorrhage, (ii) traumatic rupture, and (iii) bilateral AML. A literature search was performed using the PubMed databases to confirm this observation. Pairs of search terms were connected using the Boolean operator ‘AND’. The pairs were formed by combining the keywords “renal angiomyolipoma”, and “bilateral renal angiomyolipoma” with the terms “traumatic rupture”, “death”, and “autopsy”. In the first selection round, we included peer-reviewed publications written in English. A total of 537 articles were found. After duplicate removal, we obtained 512 articles. Two authors selected the publications for inclusion based on their titles and abstracts, removing 466 articles. In a second full-text review of the remaining 46 articles, the 3 features above were not found in a unique case. Of the 46 articles, 9 had some of our case characteristics. An extensive study of these cases helped better understand the present case’s unicity.

Regarding the bilaterality, renal angiomyolipoma is a benign tumor most frequently affecting a single kidney.^20^ Bilateral renal angiomyolipoma is relatively rare and is generally associated with a diagnosis of tuberous sclerosis.^21^ As stated by Ciancio et al.,^22^ individuals with tuberous sclerosis develop renal angiomyolipomas in 40-80% of cases, and these tend to be larger, bilateral, and more frequently symptomatic compared to those diagnosed in individuals without tuberous sclerosis. Bechtold^23^ also highlighted the rarity of bilateral renal angiomyolipoma.

Regarding renal angiomyolipoma traumatic rupture, the literature revealed three cases of patients with unilateral renal angiomyolipoma.^24-26^ All three of these cases involved women who were successfully treated. One case^27^ with bilateral renal angiomyolipoma showed a traumatic rupture successfully treated surgically.

One case,^28^ of our research sample, involved a patient with bilateral renal angiomyolipoma rupture who died after 94 days of surgical complications.

The prognosis for patients with hemorrhagic shock following renal angiomyolipoma rupture o depends on the severity of the bleeding, the tumor size and location, the presence of coagulation disorders, and the timeliness and effectiveness of treatment. Most often, after spontaneous or traumatic rupture, the patients survive after bleeding management.^29^ Cases of hemorrhagic shock have been reported successfully treated with nephrectomy.^30,31^

Patients with polytrauma and massive bleeding present a high risk of metabolic acidosis, hypothermia, and coagulopathy. In these patients, fluid resuscitation, blood transfusion, coagulopathy correction, and hypothermia prevention must be promptly introduced. Damage control surgery involving sutures and tamponades is necessary to control bleeding and resuscitation.

## CONCLUSIONS

Similar cases with rapid fatal outcomes from hemorrhagic shock due to the traumatic rupture of a renal angiomyolipoma are rare in the literature. No such fatal outcomes have been observed in cases involving the rupture of bilateral renal angiomyolipomas. The case presented aims to contribute to the discussion on the prevention and treatment of angiomyolipoma ruptures, helping to identify and prevent sensitive cases that could result in death. This report documents a rare and peculiar instance of bilateral renal angiomyolipoma leading to fatal hemorrhagic shock after a traumatic rupture. While such cases are uncommon, their documentation is vital for enriching medical knowledge and understanding of AML's clinical spectrum. Though clinically infrequent, the occurrence is a valuable academic addition, illustrating the diverse and sometimes unexpected presentations of renal angiomyolipomas. This case reinforces the importance of detailed post-mortem examinations in elucidating unusual causes of death, particularly in the context of trauma, thereby contributing to the broader understanding of AML's potential manifestations.

## References

[1] Unlü C, Lamme B, Nass P, Bolhuis HW (2006). Retroperitoneal haemorrhage caused by a renal angiomyolipoma. Emerg Med J.

[2] Steiner MS, Goldman SM, Fishman EK, Marshall FF (1993). The natural history of renal angiomyolipoma. J Urol.

[3] Jinzaki M, Silverman SG, Akita H, Nagashima Y, Mikami S, Oya M (2014). Renal angiomyolipoma: a radiological classification and update on recent developments in diagnosis and management. Abdom Imaging.

[4] Oesterling JE, Fishman EK, Goldman SM, Marshall FF (1986). The management of renal angiomyolipoma. J Urol.

[5] Corr P, Yang WT, Tan I (1994). Spontaneous haemorrhage from renal angiomyolipomata. Australas Radiol.

[6] Albi G, del Campo L, Tagarro D (2002). Wünderlich’s syndrome: causes, diagnosis and radiological management. Clin Radiol.

[7] Bissler JJ, Kingswood JC (2004). Renal angiomyolipomata. Kidney Int.

[8] Eble JN (1998). Angiomyolipoma of kidney. Semin Diagn Pathol.

[9] Lorin de la Grandmaison G, Clairand I, Durigon M (2001). Organ weight in 684 adult autopsies: new tables for a Caucasoid population. Forensic Sci Int.

[10] Lemaitre L, Claudon M, Dubrulle F, Mazeman E (1997). Imaging of angiomyolipomas. Semin Ultrasound CT MR.

[11] Lane BR, Aydin H, Danforth TL (2008). Clinical correlates of renal angiomyolipoma subtypes in 209 patients: classic, fat poor, tuberous sclerosis associated and epithelioid. J Urol.

[12] Williamson B, Bernard FK, Pollack HM, Mcclennan BL (2000). Clinical urography..

[13] Johnson SR, Cordier JF, Lazor R (2010). European Respiratory Society guidelines for the diagnosis and management of lymphangioleiomyomatosis. Eur Respir J.

[14] Avila NA, Kelly JA, Chu SC, Dwyer AJ, Moss J (2000). Lymphangioleiomyomatosis: abdominopelvic CT and US findings. Radiology.

[15] Yu DS, Wu CJ, Chang SY (2001). Growth pattern of renal angiomyolipoma on computed tomography: report of two cases. J Formos Med Assoc.

[16] van Baal JG, Smits NJ, Keeman JN, Lindhout D, Verhoef S (1994). The evolution of renal angiomyolipomas in patients with tuberous sclerosis. J Urol.

[17] Redkar N, Patil MA, Dhakate T, Kolhe P (2012). uberous sclerosis complex presenting as bilateral large renal angiomyolipomas. BMJ Case Rep.

[18] Ali S, Lalani AS, Mukherjee D, Hashmi KN (2023). A rare case of bilateral renal angiomyolipoma: radiological findings of tuberous sclerosis complex. Clin Case Rep.

[19] Heidari R, Ghadamzadeh M, Bahardoust M (2022). Association of CT scan parameters with the risk of renal angiomyolipoma rupture: a brief report. Arch Acad Emerg Med.

[20] Shah PJ, Gaches CG (1980). Bilateral renal angiomyolipoma: a case report. Eur Urol.

[21] Tongaonkar HB, Sampat MB, Dalal AV, Dandekar NP, Kulkarni JN, Kamat MR (1994). Bilateral renal angiomyolipoma. J Surg Oncol.

[22] Ciancio SJ, Vira M, Simon MA, Lerner SP, Schulam PG (2001). Giant bilateral renal angiomyolipomas associated with tuberous sclerosis. Urology.

[23] Bechtold IR (1976). Multiple bilateral renal angiomyolipoma: case report. Scand J Urol Nephrol.

[24] Lai CC, Fan WC, Chao CM, Liu WL, Hou CC (2012). Traumatic rupture of a renal angiomyolipoma. J Emerg Med.

[25] Renz B, Sorini P, Wachtel TL, Perry R (1989). Traumatic rupture of a renal angiomyolipoma. Injury.

[26] Beh WP, Barnhouse DH, Johnson SH, Marshall M, Price SE (1976). A renal cause for massive retroperitoneal hemorrhage: renal angiomyolipoma. J Urol.

[27] Tsai CK, Lin YT, Lin TC (2010). Traumatic rupture of bilateral huge renal angiomyolipomas in tuberous sclerosis complex. J Trauma.

[28] Mantas D, Papachristodoulou A (2006). Bilateral renal angiomyolipoma. Acta Chir Belg.

[29] Faraji H, Nguyen BN, Mai KT (2009). Renal epithelioid angiomyolipoma: a study of six cases and a meta-analytic study. Development of criteria for screening the entity with prognostic significance. Histopathology.

[30] Wang H-B, Yeh C-L, Hsu K-F (2009). Spontaneous rupture renal angiomyolipoma with hemorrhagic shock. Intern Med.

[31] Cho E, Morozumi M, Yano A (2017). Huge renal angiomyolipoma complicated with common iliac vein thrombus because of the tumor pressure. Nippon Hinyokika Gakkai Zasshi.

